# Mechanical Tension Increases CCN2/CTGF Expression and Proliferation in Gingival Fibroblasts via a TGFβ-Dependent Mechanism

**DOI:** 10.1371/journal.pone.0019756

**Published:** 2011-05-17

**Authors:** Fen Guo, David E. Carter, Andrew Leask

**Affiliations:** 1 Department of Dentistry, University of Western Ontario, London, Ontario, Canada; 2 London Regional Genomics Centre Microarray Facility, Robarts Research Institute, London, Ontario, Canada; 3 Department of Physiology and Pharmacology, University of Western Ontario, London, Ontario, Canada; Université de Technologie de Compiègne, France

## Abstract

Unlike skin, oral gingival do not scar in response to tissue injury. Fibroblasts, the cell type responsible for connective tissue repair and scarring, are exposed to mechanical tension during normal and pathological conditions including wound healing and fibrogenesis. Understanding how human gingival fibroblasts respond to mechanical tension is likely to yield valuable insights not only into gingival function but also into the molecular basis of scarless repair. CCN2/connective tissue growth factor is potently induced in fibroblasts during tissue repair and fibrogenesis. We subjected gingival fibroblasts to cyclical strain (up to 72 hours) using the Flexercell system and showed that CCN2 mRNA and protein was induced by strain. Strain caused the rapid activation of latent TGFβ, in a fashion that was reduced by blebbistatin and FAK/src inhibition, and the induction of endothelin (ET-1) mRNA and protein expression. Strain did not cause induction of α-smooth muscle actin or collagen type I mRNAs (proteins promoting scarring); but induced a cohort of pro-proliferative mRNAs and cell proliferation. Compared to dermal fibroblasts, gingival fibroblasts showed reduced ability to respond to TGFβ by inducing fibrogenic mRNAs; addition of ET-1 rescued this phenotype. Pharmacological inhibition of the TGFβ type I (ALK5) receptor, the endothelin A/B receptors and FAK/src significantly reduced the induction of CCN2 and pro-proliferative mRNAs and cell proliferation. Controlling TGFβ, ET-1 and FAK/src activity may be useful in controlling responses to mechanical strain in the gingiva and may be of value in controlling fibroproliferative conditions such as gingival hyperplasia; controlling ET-1 may be of benefit in controlling scarring in response to injury in the skin.

## Introduction

Fibrosis is the formation of excessive connective tissue in a reparative or reactive process. Scars are areas of fibrosis that replace normal tissue after injury; excessive scarring can obliterate tissue architecture, culminating culminate in organ failure and death. Scarring in response to wounding occurs in skin, but not in the oral cavity [1]. Fibroblasts, which are embedded within connective tissue, are the cell type responsible for connective tissue repair and fibrosis [2], and thus it is reasonable to hypothesize that differences in response of gingival and dermal fibroblasts to fibrogenic stimuli are likely to underlie the basis of scarless tissue repair.

One such fibrogenic stimulus may be mechanical tension. Although most tissues exist under a mechanical tension, resident fibroblasts are normally ‘stress-shielded’ by the matrix that they deposit and remodel and thus are protected from the external loads by the mechanical properties of the surrounding matrix; however, this protection is lost during injury [3]. Indeed, fibroblasts are subjected to alterations in mechanical during physiological as well as pathological situations, such as wound healing, development of hypertrophic scars, and fibrogenesis. The effect of mechanical forces on gene regulation have been mainly studied in endothelial and smooth muscle cells or chondrocytes that are constantly subjected to high fluid shear or pressure loading [4]; however, one study showed that application of strain to dermal fibroblasts resulted in their differentiation of myofibroblasts, as visualized by the induction of collagen type I and α-smooth muscle actin (α-SMA) [5], the key cell type responsible for scarring [6]. Conversely, the responses of gingival fibroblasts to mechanical loading are almost wholly unknown, which is perhaps surprising since orthodontic forces are constantly affecting the extracellular matrix (ECM) and the cells within dental pulp, periodontal ligament, alveolar bone, and gingiva [7]–[9]. Indeed, it has been hypothesized that application of external mechanical loads to teeth may alter the forces acting on gingival fibroblasts, leading to changes in gene expression ultimately culminating in alteration in the structure and function of the ECM [7]; however, this hypothesis has yet to be tested. Thus understanding how gingival fibroblasts respond to mechanical loading is therefore necessary to not only understand how these cells respond to normal orthodontic forces, but may also reveal valuable insights into the potential molecular basis of scarless tissue repair.

The protein connective tissue growth factor (CTGF/CCN2), a member of the CCN (Cyr61, ctgf, nov) family of matricellular proteins, is potently induced by fibrogenic protein transforming growth factor (TGF)β [10], [11]. CCN2 expression correlates well with the onset of tissue repair and fibrotic conditions, including those affecting the oral cavity such as phenytoin-induced gingival overgrowth or hereditary gingival fibromatosis [10]–[15], and appears to contribute to collagen deposition in these processes; for example, mice deficient in CCN2 expression in dermal fibroblasts are resistant to bleomycin-induced skin fibrosis [16]. CCN2 has been shown to respond to mechanical strain in a bladder and endothelial cells [17], [18], but whether CCN2 is induced in response to strain in gingival fibroblasts is not known. Examining the control of CCN2 expression in response to strain in gingival fibroblasts is therefore likely to represent a useful tool in understanding the molecular mechanism underlying the ability of strain to modulate gene expression in gingival fibroblasts.

In this report, we investigate of mechanical strain on gene expression in gingival fibroblasts by monitoring the alterations in (a) CCN2 expression using real-time polymerase chain reaction and Western blot analyses and (b) genome-wide mRNA expression using micro-array profiling. Our results not only give new and valuable insights into the molecular mechanism underlying how gingival fibroblasts respond to strain and but may also have long-term consequences for understanding how to modulate gene expression in pathological conditions such as gingival hyperplasia and also in understanding the potential mechanism underlying scarless repair in the oral cavity.

## Methods

### Cell Culture

Gingival fibroblasts from three human donors (HGF) were used for this study and were identical to those previously described [14]. Cells were cultured in DMEM, supplemented with 10% fetal bovine serum, 1% antibiotic-antimycotic (Invitrogen), in a humidified 5% CO_2_ at 37°C. All experiments were performed on cells between passage 5 and 7. Human dermal fibroblasts (HDF) were purchased (ATCC) and cultured identically.

### Mechanical stress

HGF (1×10^5^) were cultured on 6 well plates with a flexible, silicon-based well that was coated with type I collagen (Flexcell), and then subjected to 10% uniaxial cyclic strain at 0.5 Hz for up to 72 hours using a Flexercell apparatus (Flexcell). Parameters were chosen based on their ability to maximally induce CCN2 mRNA. Controls were prepared in an identical manner and cultured on unstrained type I collagen-coated plates. For inhibitors treatment, hGF were rendered quiescent by 24-hour incubation in DMEM with 0.5% FBS. Inhibitors of the TGFβ type I (ALK5) receptor (SB431542; 10 µM; please note that this inhibitor also affects the activin type I receptor ALK4 and the nodal type I receptor ALK7), FAK/src (PP2; 10 µM), ETA/B receptor (PD145065; 10 µM), the actin/myosin destabilizing agent blebbistatin (12.5 µM) (all purchased from Calbiochem) or DMSO control was then added to the culture medium before subjected to mechanical stretch. These concentrations of inhibitors have been previously shown to be effective and selective for their respective targets [19]–[24]. When indicated, conditioned culture supernatants or cell layers were isolated for protein or mRNA analysis.

### TGFβ1 stimulation

HGF and HDF cells were cultured on 6 well plates (plastic, Greiner Bio-one) until 60% confluent, serum-starved (DMEM, 0.5% FBS) for 24 hours and then treated with or without TGFβ1 (4 ng/ml; R and D Systems) for an additional 6 hours prior to RNA extraction. For rescue experiment, cells were similarly cultured and preincubated with or without endothelin-1 (ET-1, 100 nM; R and D Systems) for 30 min prior to the incubation with or without TGFβ1 for additional 6 hours.

### Real-time polymerase chain reaction

Real-time PCR was performed as previously described [14], [25]. Total RNA was isolated (Trizol, Invitrogen) and then was reverse transcribed and amplified using TaqMan Assay-on-Demand (Applied Biosystems) in a 15-µl reaction volume containing 2 unlabeled primers and a 6-FAM–labeled TaqMan minor groove binder. Samples were combined using One-Step Master Mix (Applied Biosystems), and amplified sequences were detected using the ABI Prism 7900HT Sequence Detector (Perkin-Elmer-Cetus) according to the manufacturer's instructions. Triplicate samples were run. Expression values were standardized to values obtained with control 18S RNA primers, using the 2-^ΔΔCt^ method.

### ELISA assays

The concentration of active TGFβ1 in the cell culture supernatants was measured using TGFβ1 Emax® ImmunoAssay System (Promega). For active TGFβ1, 100 µl culture supernatants were used in the Emax immunoassay, which was performed according to the manufacture's instructions [26]. The standard curve is linear between 15.6 and 1,000 pg/ml of the TGFβ1 standard. TGFβ1 standard curves were undertaken for every assay. All experiments were performed in triplicate. The data are represented as the mean values of these triplicate samples.

The secreted endothelin-1 (ET-1) level in the culture supernatants was determined in triplicate using a Quantiglo Human Endothelin-1 Immunoassay (R&D Systems). 100 µl culture supernatants were used in the Quantiglo immunoassay, which was performed according to the manufacturer's instructions. The standard curve is linear between 0.34 and 250 pg/ml of the endothelin-1 standard and was conducted for every assay.

### Western Blotting

Samples containing 100 µg of protein were subjected to SDS-PAGE and then transferred to PVDF membranes (Invitrogen). The membranes were blocked with 5% milk-TBST for 1 hour at room temperature, incubated with anti-CCN2 antibody (Santa Cruz, 1∶200 dilution) overnight at 4°C, washed with TBST, incubated with secondary goat anti-mouse antibody (Jackson Immunoresearch, 1∶10000) conjugated to horseradish peroxidase, washed and visualized with ECL Western Blotting Detection Reagents (Amersham Biosciences). After stripping with Restore Western Blot Stripping Buffer (Pierce) for 20 minutes at room temperature, membranes were processed similarly with β-actin antibody (Sigma, 1∶10000 dilution) as a loading control.

### Expression Profiling

Expression profiling was conducted essentially as previously described [27], [28]. All sample labeling and GeneChip processing was performed at the London Regional Genomics Centre (Robarts Research Institute, London, Ontario, Canada; http://www.lrgc.ca). RNA quality was assessed using the Agilent 2100 Bioanalyzer (Agilent Technologies Inc., Palo Alto, CA) and the RNA 6000 Nano kit (Caliper Life Sciences, Mountain View, CA). Single stranded complimentary DNA (sscDNA) was prepared from 200 ng of total RNA as per the Ambion WT Expression Kit for Affymetrix GeneChip Whole Transcript WT Expression Arrays (http://www.ambion.com/techlib/prot/fm_4411973.pdf, Applied Biosystems, Carlsbad, CA) and the Affymetrix GeneChip WT Terminal Labeling kit and Hybridization User Manual (http://media.affymetrix.com/support/downloads/manuals/wt_term_label_ambion_user_manual.pdf, Affymetrix, Santa Clara, CA). Total RNA was first converted to cDNA, followed by *in vitro* transcription to make cRNA. 5.5 µg of single stranded cDNA was synthesized, end labeled and hybridized, for 16 hours at 45°C, to Human Gene 1.0 ST arrays. All liquid handling steps were performed by a GeneChip Fluidics Station 450 and GeneChips were scanned with the GeneChip Scanner 3000 7G (Affymetrix, Santa Clara, CA) using Command Console v1.1. Probe level (.CEL file) data was generated using Affymetrix Command Console v1.1. Probes were summarized to gene level data in Partek Genomics Suite v6.5 (Partek, St. Louis, MO) using the RMA algorithm. Partek was used to determine gene level ANOVA p-values, fold changes and GO (Gene Ontology) enrichment, using a Chi squared test. Experiments were performed twice, and fold changes were identified using the GeneSpring filter. The fold change between with and without mechanical strain treatment had to be at least 1.5 fold to identify a transcript as being altered (p<0.05) and these genes list was compiled and exported into DAVID (http://david.abcc.ncifcrf.gov/) for further analysis.

### Proliferation assay

Gingival fibroblasts were seeded on type I collagen-coated plate at a density of 50,000 cells per well. After 24 hour of serum-starvation (DMEM, 0.5% FBS), DMSO or TGFβ type I (ALK5) receptor (SB431542; 10 µM), FAK/src (PP2; 10 µM) or ETA/B receptor (PD145065; 10 µM) inhibitor was added to the culture medium. Cells were then subjected to 10% uniaxial cyclic strain at 0.5 Hz for 72 hours using a Flexercell apparatus (Flexcell). Controls were prepared in the same way and cultured on an unstrained type I collagen-coated plate. After 72 hours stretch, cells underwent evaluation for proliferation using an MTT [3-(4,5-dimethylthiazol-2-yl)-2,5-diphenyltetrazolium bromide] assay, which indirectly measures cell proliferation by assessing metabolic activity, as described by the manufacturer (Roche). Briefly, cells were washed twice with PBS, and l.5 ml of serum-free medium containing 100 µg/ml MTT was added to each well. After incubation at 37°C for 4 h, 1.5 ml solubilization solution was added to dissolve MTT crystals overnight. Two hundred microlitres of solution from each well were transferred into 96-well microplate and measured for absorbance at 570 nm with reference at 680 nm.

### Statistical Analysis

Statistical tests were done using one-way ANOVA analysis of variance and Tukey's *post hoc* test with GraphPad Software V.4 (Graphpad Software, La Jolla, CA, USA). P values less than 0.05 were taken to be significant.

## Results

### CCN2/CTGF mRNA and protein are induced in response to strain in human gingival fibroblasts

**Figure 1:**
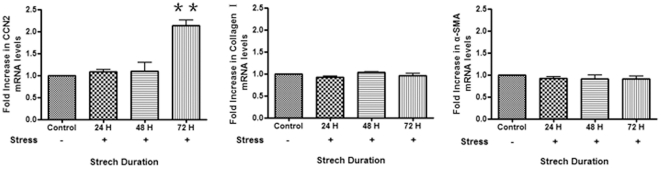
Mechanical strain induces CCN2/CTGF mRNA expression in human gingival fibroblasts (HGF). As described in Methods, equal numbers of HGF were seeded into plates containing collagen type I-coated membranes and subjected to the presence or absence of mechanical strain for up to 72 hours. Total RNA was harvested, and subjected to real-time PCR analysis with primers detecting CCN2, collagen type I (Col1a2) or α-SMA. Expression values are adjusted to those of controls (18S) run in parallel. Experiments were performed on cells derived from three different individuals, with quadruplicate replicate samples. Relative expression at time zero (control) was taken to represent 1. Fold increase in relation to time zero (control) is shown. Data shown are the average value from three independent individuals (3 replicates *per experiment*) ±SE. ** = p<0.01. Note that α-SMA and Col1a2 mRNAs were not significantly induced by strain. Similar results were obtained if cells were plated on laminin-coated membranes (not shown).

Because of the known utility of CCN2 as a molecular marker of tissue repair and fibrogenic responses [10], [15], we were initially interested in examining whether mechanical strain could induce CCN2 mRNA expression in gingival fibroblasts. To perform this analysis, we used the Flexercell system, employing 6-well tissue culture plates possessing silicon membranes coated with type I collagen. Equal numbers of cells were placed on tissue culture plates and treated with or without strain (10% uniaxial cyclic strain, 0.5 Hz) for up to 72 hours. Cells from three separate individuals were used for our analyses. Real time PCR analysis of total RNA extracted from these cells revealed that, after application of strain for 72 hours, CCN2 mRNA was statistically significantly in cells subjected to strain, relative to cells that were not subjected to strain for the same duration (Fig. 1). Similar results when cells were cultured on laminin-coated plates (not shown). Intriguingly, mRNA expression of neither collagen type I (Col 1a2) nor α-SMA was altered by strain (Fig. 1).

### Strain results in elevated TGFβ activity and expression in gingival fibroblasts

**Figure 2:**
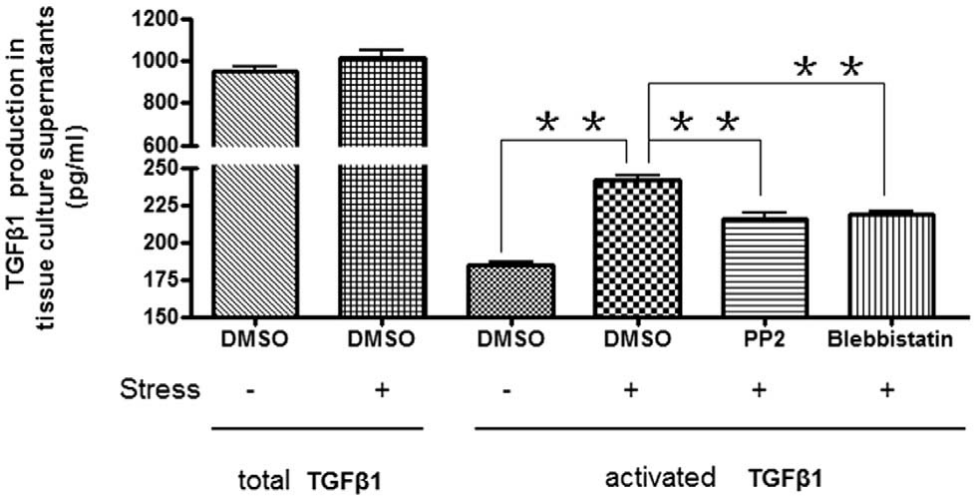
Strain induces the activation of latent TGFβ in human gingival fibroblasts. As described in Methods, equal numbers of HGF were seeded into plates containing collagen type I-coated membranes, and were treated with or without mechanical strain for 24 hours. As described in methods, ELISA was used to detect total and activated TGFβ in the presence or absence of DMSO, blebbistatin (which blocks activation of TGFβ by impairing actin/myosin-dependent cell contraction [24], [29], [30]) or the FAK/src inhibitor PP2. Note induction of active TGFβ at the 24 hour time-point in the presence of strain and DMSO relative to cells treated with blebbistatin, strain and PP2, and control cells not subjected to strain. Data shown are the average value from three independent individuals (3 replicates *per experiment*) ±SE. ** = p<0.01.

The kinetics of CCN2 induction in response to mechanical stain suggested that strain indirectly resulted in increased CCN2 expression (Fig. 1). Contraction of ECM by skin and lung fibroblasts causes activation of latent TGFβ via integrins; this activation is blocked by the actin/myosin destabilizing agent blebbistatin which blocks ECM contraction by fibroblasts [24], [29], [30]. Based on these observations, we hypothesized that mechanical strain could elevate CCN2 mRNA and protein in gingival fibroblasts indirectly via the ability of strain to induce activation of TGFβ. To begin to investigate this hypothesis, we used specific ELISAs to show that strain (24 hours) could induce the appearance of activated TGFβ; total TGFβ levels were unaltered (Fig. 2). This increase in activated TGFβ was significantly reduced by the actin/myosin depolymerizing agent blebbistain (Fig. 2). Focal adhesions mediate the interaction of integrins with the ECM; inhibition of focal adhesion kinase (FAK)/src with PP2 (10 µM) significantly reduced the ability of strain to induce activation of TGFβ (Fig. 2). Collectively, these data suggest the involvement of contraction in this process.

### CCN2/CTGF is induced in response to strain via a TGFβ type I receptor (ALK5)-dependent mechanism

**Figure 3:**
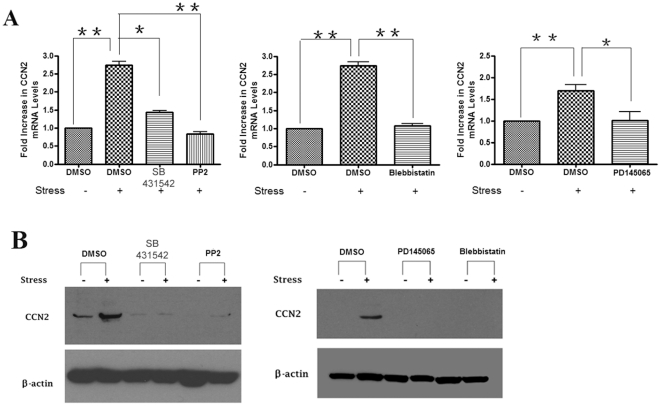
The ability of strain to induce CCN2 expression in blocked by ALK5 or ETA/B receptor inhibition. As described in Methods, equal numbers of HGF were seeded into plates containing collagen type I-coated membranes, and were treated with or without mechanical strain for 72 hours. Cells were treated with or without DMSO, the ALK5 inhibitor SB431542, PP2, blebbistatin or the ETA/B receptor antagonist PD145065, as indicated. (A) Total RNA was harvested, and subjected to real-time PCR analysis with primers detecting CCN2. Expression values are adjusted to those of controls (18S) run in parallel. Experiments were performed on cells derived from three different individuals, with quadruplicate replicate samples. (B) Cells were treated with or without DMSO, the ALK5 inhibitor SB431542, PP2, blebbistatin or the ETA/B receptor antagonist PD145065, as indicated. Total protein was harvested, and subjected to Western blot analysis with anti-CCN2 or anti-β-actin antibodies, as indicated. Data shown are the average value from three independent individuals (3 replicates *per experiment*) ±SE. * = p<0.05; ** = p<0.01.

To address whether the increase in CCN2 expression in response to strain was dependent on TGFβ, we initially performed real-time PCR analysis on mRNAs treated with or without mechanical strain for 72 hours. This time point was chosen as previously we had determined that CCN2 mRNA was induced 72 hours-post-strain, but not at earlier timepoints (Fig. 1). We found that inhibition of the TGFβ type I (ALK5) receptor (SB431542; 10 µM) or FAK/src (PP2; 10 µM) significantly blocked the ability of strain to induce CCN2 mRNA and protein in gingival fibroblasts (Fig. 3A, B). Moreover, consistent with the notion that loading/contraction-mediated activation of TGFβ resulted in the elevation of CCN2 expression, blebbistatin reduced the ability of strain to induce CCN2 mRNA and protein in gingival fibroblasts (Fig. 3A, B). Collectively, our data suggest that exposure of cyclic mechanical strain to gingival fibroblasts indirectly causes the induction of CCN2 through increased activation of latent TGFβ.

### The ability of strain to induce CCN2 depends on signaling through the endothelin A/B (ETA/B) receptors

**Figure 4:**
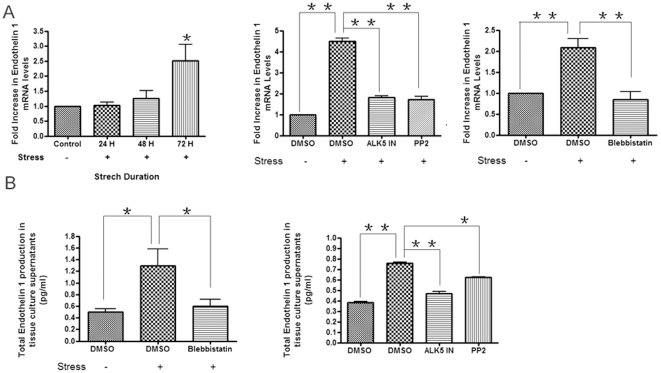
Strain induces endothelin-1 (ET-1) production. As described in Methods, equal numbers of HGF were seeded into plates containing collagen type I-coated membranes, and were treated with or without mechanical strain for 72 hours. Cells were treated with or without DMSO, the ALK5 inhibitor SB431542, PP2, or blebbistatin, as indicated. (A) Total RNA was harvested, and subjected to real-time PCR analysis with primers detecting ET-1. Expression values are adjusted to those of controls (18S) run in parallel. Experiments were performed on cells derived from three different individuals, with quadruplicate replicate samples. (B) Conditioned media was subjected to ELISA to detect ET-1. Data shown are the average value from three independent individuals (3 replicates *per experiment*) ±SE. * = p<0.05; ** = p<0.01.

Endothelin-1 (ET-1), which signals through the ETA/B receptors, is a potent vasoconstrictory peptide that can act both downstream and concomitant with TGFβ to activate fibrogenic gene expression [31]–[33]. To assess whether ET-1 was involved with the ability of strain to induce CCN2, we first used real-time PCR and a specific ELISA to show that strain induced ET-1 mRNA (Fig. 4A) and protein (Fig. 4B) expression in a fashion that was sensitive to ALK5 inhibition. Moreover, consistent with the notion that loading/contraction-mediated activation of TGFβ resulted in the elevation of endothelin-1 expression, blebbistatin and PP2 reduced the ability of strain to induce ET-1 mRNA and protein in gingival fibroblasts (Fig. 4A, B). Having shown that strain could induce ET-1, we then used real time PCR and Western blot analyses to show that the ETA/B receptor inhibitor PD145065 (10 µM) could significantly reduce the ability of strain to elevate CCN2 mRNA and protein levels (Figs. 3A, B). These data suggest that strain induces CCN2 production in a fashion dependent on (a) the ability of strain to activate latent TGFβ via a actin/myosin and FAK/src dependent mechanism (contraction) and (b) on the ability of TGFβ to elevate ET-1 production.

### Gingival fibroblasts are less responsive to TGFβ than dermal fibroblasts; addition of ET-1 to gingival fibroblasts rescues this phenotype

**Figure 5:**
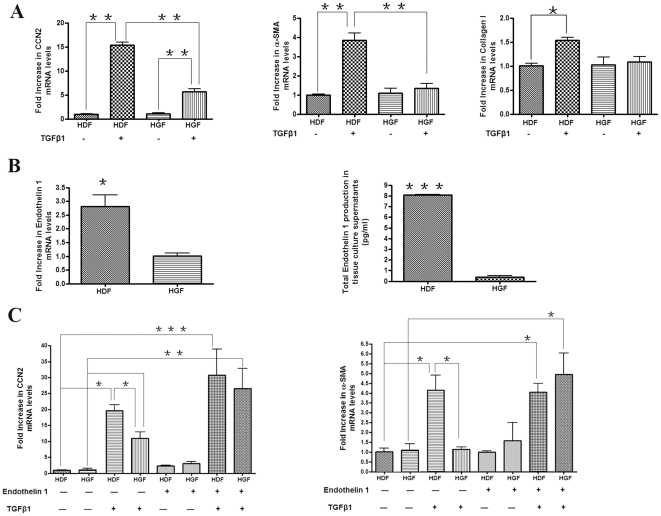
Human gingival fibroblasts are less sensitive to TGFβ1 than human dermal fibroblasts and addition of ET-1 rescued this phenotype. (A) Equal numbers of human gingival fibroblasts (HGF) and human dermal fibroblasts (HDF) of equal passage were plated onto tissue culture plates (plastic) and treated with or without TGFβ1 (4 ng/ml) for 6 hours. Total RNA was harvested, and subjected to real-time PCR analysis with primers detecting CCN2, collagen type I (Col1a2) or α-SMA. Expression values are adjusted to those of controls (18S) run in parallel. (B) HGF and HDF cells were plated onto tissue culture plates (plastic), both cells and cell culture supernatants were harvested. Total RNA were extracted and subjected to real-time PCR analysis with primers detecting ET-1. Expression values are adjusted to those of controls (18S) run in parallel. Cell culture supernatants were subjected to ELISA to detect ET-1. (C) As described in Methods, cells were preincubated with or without Endothelin 1 (100 nM; R and D Systems) for 30 min prior to the incubation with or without TGFβ1 for additional 6 hours. Total RNA was harvested, and subjected to real-time PCR analysis with primers detecting CCN2, collagen type I (Col1a2) or α-SMA. Expression values are adjusted to those of controls (18S) run in parallel. Data shown are the average value from three independent individuals (3 replicates *per experiment*) ±SE. * = p<0.05; ** = p<0.01; *** = p<0.001.

It was interesting that whereas mechanical strain induced TGFβ activation, ET-1 and CCN2 and TGFβ mRNAs, but not type I collagen or αSMA mRNAs, were induced. This result suggested the intriguing possibility that gingival fibroblasts were relatively unable to respond to TGFβ by inducing type I collagen or αSMA mRNAs. To further explore this notion, we compared the responses of gingival and dermal fibroblasts to TGFβ1. We showed that TGFβ1 (4 ng/ml, 6 hours) caused potent induction of CCN2, collagen I and αSMA mRNAs in human dermal fibroblasts (Fig. 5A). Note that of these mRNAs, CCN2 mRNA was the transcript that was most highly induced by TGFβ1. Conversely, human gingival fibroblasts were less responsive to TGFβ1 (4 ng/ml, 6 hours). These results indicate that the basis for the inability of strain to induce type I collagen and α-SMA mRNAs was likely to arise due to the relative inability of gingival fibroblasts to respond to TGFβ by inducing these mRNAs.

Since ET-1 can synergize with TGFβ [32], we began to investigate whether the relative inability of gingival fibroblasts to respond to TGFβ could be due to diminished ET-1 production. We found that, both at an mRNA and a protein level, gingival fibroblasts showed reduced ET-1 production (Fig. 5B). The key feature of fibrotic cells is differentiation of fibroblasts to α-SMA-expressing myofibroblasts [2], [3]; addition of recombinant ET-1 to gingival fibroblasts rescued the ability of TGFβ to potently induce CCN2 and α-SMA mRNAs in gingival fibroblasts (please note that the relatively modest response of dermal fibroblasts by inducing collagen mRNA precluded the use of this transcript in our rescue experiment) (Fig. 5C).

### Mechanical strain induces the proliferation of gingival fibroblasts

**Figure 6:**
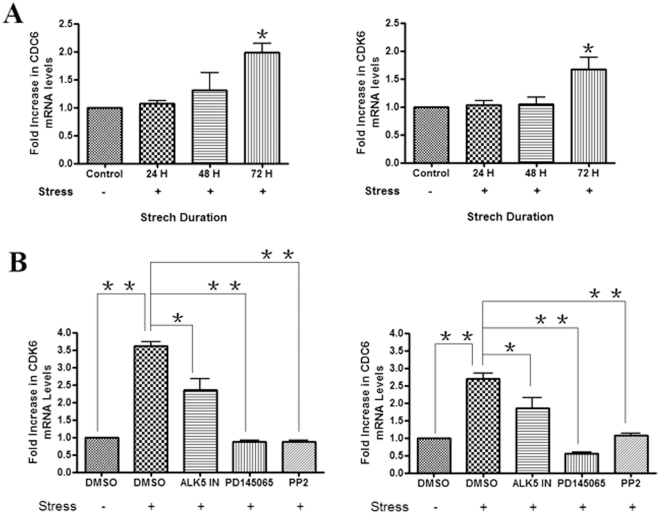
Strain induces cdc6 and cdk6 mRNAs in human gingival fibroblasts. (A) As described in Methods, equal numbers of HGF were seeded into plates containing collagen type I-coated membranes, and were treated with or without mechanical strain for up to 72 hours. Total RNA was harvested, and subjected to real-time PCR analysis with primers detecting cdc6 and cdk6 mRNAs. Expression values are adjusted to those of controls (18S) run in parallel. (B) Experiments similar to those performed in (A) were conducted with or without mechanical strain (72 hours) in the presence or absence of DMSO, the ALK5 inhibitor SB431542, PP2, blebbistatin or the ETA/B receptor antagonist PD145065, as indicated. Total RNA was harvested, and subjected to real-time PCR analysis with primers detecting cdc6 and cdk6 mRNAs. Expression values are adjusted to those of controls (18S) run in parallel. Data shown are the average value from three independent individuals (3 replicates *per experiment*) ±SE. * = p<0.05; ** = p<0.01.

**Figure 7:**
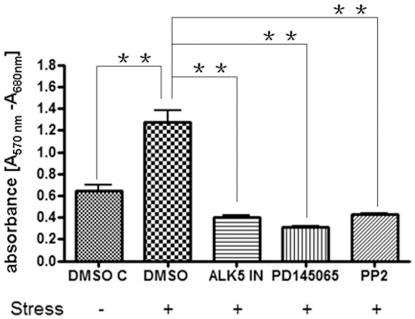
Strain induces proliferation of human gingival fibroblasts. As described in Methods, equal numbers of HGF were seeded into plates containing collagen type I-coated membranes, and were treated with or without mechanical strain for 72 hours. Cells were harvested, and subjected to the MTT assay of cell metabolism, an indirect method of detecting cell proliferation, as described in methods. Cells were treated in the presence or absence of DMSO, the ALK5 inhibitor SB431542, PP2, blebbistatin or the ETA/B receptor antagonist PD145065, as indicated. Data shown are the average value from three independent individuals (6 replicates *per experiment*) ±SE. ** = p<0.01.

Given that gingival fibroblasts clearly were able to respond to strain, as visualized by their ability to support CCN2, TGFβ and ET-1 induction, we then used genome-wide expression profiling to investigate the overall effect of mechanical strain on gene regulation in gingival fibroblasts. Thus, genome-wide expression profiling was conducted on gingival fibroblasts treated with or without mechanical strain for 72 hours. RNAs were extracted, and subjected to Affymetrix gene profiling and cluster analysis. No ‘fibrotic’ cluster was identified; however, a cluster containing genes involved with cell proliferation was found to be induced in response to strain (Table 1). The mRNAs encoding cdc6 and cdk6 were selected for further analysis. Cell division cycle 6 (CDC6) is an essential regulator of DNA replication in eukaryotic cells [34] and cyclin-dependent kinases (including cdk6) are catalytic subunits of a family of mammalian heterodimeric serine/threonine kinases implicated in the control of cell-cycle progression [35]. Thus these mRNAs were selected for further analysis. Real time PCR analysis verified our gene array data that cdc6 and cdk6 were induced in response to strain (Fig. 6A). Moreover, the induction of cdc6 and cdk6 mRNAs was reduced by ALK5, ETA/B and FAK/src inhibition (Fig. 6B). Providing a functional context to our studies, we found that mechanical strain (72 hours) induced proliferation of gingival fibroblasts in a fashion which was sensitive to ALK5, ETA/B and FAK/src inhibition (Fig. 7).

**Table 1:** Cluster analysis of mRNA (out of 453 total) induced more than 1.5-fold by mechanical stress. Average expression value is shown. (P<0.05).

Affymetrix ID	RefSeq	Gene name		Fold up( Stress VS Contol)
*regulation of cell cycle*				
8017262	NM_032043	BRCA1 interacting protein C-terminal helicase 1	BRIP1	2.49846
7986068	NM_000057	Bloom syndrome, RecQ helicase-like	BLM	2.47173
8112327	NM_001826	CDC28 protein kinase regulatory subunit 1B	CKS1B	1.94192
8071212	NM_003504	CDC45 cell division cycle 45-like	CDC45L	1.80526
8065710	NM_005225	E2F transcription factor 1	E2F1	1.56671
8130374	NM_012177	F-box protein 5	FBXO5	2.55508
8073858	NM_016426	G-2 and S-phase expressed 1	GTSE1	1.64131
7952179	NM_002105	H2A histone family, member X	H2AFX	1.69245
7924096	NM_002497	NIMA (never in mitosis gene a)-related kinase 2	NEK2	2.46098
7933707	NM_032997	ZW10 interactor	ZWINT	2.09189
8132318	NM_018685	anillin, actin binding protein	ANLN	2.86885
8010260	NM_001168	baculoviral IAP repeat-containing 5	BIRC5	1.80698
7968484	NM_000059	breast cancer 2, early onset	BRCA2	1.61286
7927710	NM_001786	cell division cycle 2, G1 to S and G2 to M	CDC2	2.75341
8007071	NM_001254	cell division cycle 6 homolog	CDC6	1.623984
8102643	NM_001237	cyclin A2	CCNA2	2.28238
8105828	NM_031966	cyclin B1	CCNB1	2.33148
8151871	NM_057749	cyclin E2	CCNE2	2.547
7956076	NM_001798	cyclin-dependent kinase 2	CDK2	2.04659
8140955	NM_001259	cyclin-dependent kinase 6	CDK6	1.55686
8116921	NM_001955	endothelin 1	EDN1	1.53003
7982889	NM_016359	nucleolar and spindle associated protein 1	NUSAP1	2.29659
8063043	NM_181802	ubiquitin-conjugating enzyme E2C	UBE2C	1.58997

Collectively, these data suggest that mechanical strain induces CCN2 expression and cell proliferation via its ability to cause activation of latent TGFβ by a cell contraction (actin/myosin and FAK/src)-dependent mechanism.

## Discussion

Our manuscript is the first to show: (a) CCN2 is induced by mechanical strain in fibroblasts; (b) this induction occurs via TGFβ, ET-1 and FAK/src; (c) ET-1 is induced in fibroblasts in response to strain; (d) the transcriptional response in fibroblasts to strain depends on ET-1 and FAK/src; (e) the overall global profile of mRNAs induced in gingival fibroblasts in response to strain; (f) strain induces a series of pro-proliferative mRNAs and cell proliferation in gingival fibroblasts via TGFβ, ET-1 and FAK/src; (g) that, when applied to fibroblasts, strain does not potently induce α-SMA (a marker of myofibroblast differentiation, the key phenotypic feature of fibrotic fibroblasts [2], [3]); (h) that, when applied to fibroblasts, TGFβ is less potent at inducing CCN2 and α-SMA in gingival fibroblasts compared to dermal fibroblasts; (i) gingival fibroblasts possess reduced basal levels of ET-1 than dermal fibroblasts; and (j) application of exogenous ET-1 to gingival fibroblasts rescues the ability of TGFβ to potently induce CCN2 and α-SMA expression.

Fibrosis, the excessive production and contraction of ECM in connective tissue resulting in scarring and often organ failure and death, is one of the largest groups of diseases for which there is no therapy [36]. Embryos and gingiva tissue do not scar [1]. Although it is likely that a major cause for the differences between adult and embryonic repair, is likely to be the heightened inflammatory response that occurs in the former situation [37], it is also reasonable to posit that differences in fibroblast, the resident responder cell in connective tissue, responses to fibrogenic stimuli might also be important. In this report, we showed that gingival fibroblasts responded to strain by inducing the activation of latent TGFβ, in a fashion which was sensitive to blebbistatin and PP2 (i.e., contraction of ECM via actin/myosin and FAK/src), consistent with the prior observation using lung and dermal fibroblasts that contraction (mechanical loading) of ECM induces activation of TGFβ through an integrin/actomyosin dependent mechanism [29], [30]. However, until this report, such a mechanism has not been shown to operate in gingival fibroblasts. Moreover, the overall sequence starting from application of mechanical strain to the eliciting of phenotypic alterations in gingival fibroblasts has not been elucidated until now. As a result of strain, TGFβ, ET-1, CCN2/CTGF and proliferative mRNAs/proliferation were induced.

Fibroproliferative conditions (e.g gingival hyperplasia) are well-known to affect the gingiva and, in these conditions, CCN2/CTGF is overexpressed [10]–[15]. Moreover, we have previously shown that TGFβ induces CCN2 expression in gingival fibroblasts via ALK5 [14]. These observations are consistent with our observations that gingival fibroblasts respond to strain by inducing the expression of CCN2 as well as pro-proliferative mRNAs, and proliferation via a TGFβ-dependent mechanism. What was surprising, and in contrast to results previously obtained using dermal fibroblasts [5], were our observations that neither strain nor TGFβ potently induced the expression of α-SMA or type I collagen mRNAs. As α-SMA and type I collagen expression is a hallmark of myofibroblast differentiation and as myofibroblasts are the cell type believed to be responsible for scar tissue [2], [3], these differential responses of gingival and dermal fibroblasts may underlie the basis of scarless tissue repair in gingival fibroblasts. The precise molecular basis underlying this differential sensitivity, at least in terms of CCN2 and α-SMA mRNAs, appeared to be due to a decreased production of ET-1 by gingival fibroblasts. This observation is interesting in light of previous observations, using dermal and lung fibroblasts, that ET-1 can induce CCN2 and that ET-1 synergizes with TGFβ to induce gene expression [32], [33]. Moreover, our data illustrating that ET-1 may be a key fibrogenic molecule for fibroblasts are consistent with our previous observations that ET-1 can elicit a fibrogenic response by itself and is responsible for the persistent fibrotic phenotype of scleroderma lung fibroblasts [38]. It is interesting to note that, although strain was able to induce ET-1 in gingival fibroblasts and elicit a proliferative response via ET-1, similar to a recent report in which thrombin was shown to induce proliferation in gingival fibroblasts via ET-1 [39], the level of ET-1 generated was insufficient to potently induce CCN2 or α-SMA mRNAs.

Collectively, our results suggest that mechanical tension, a key feature or normal orthodontic forces and tissue remodeling and repair, results in the induction of active TGFβ, CCN2 and proliferative responses but not in potent activation of collagen type I or α-SMA mRNAs consistent with the notion that gingival do not scar in response to wounding. CCN2, TGFβ signaling via ALK5 or activation of latent TGFβ through ECM contraction may be useful in the future as strategies to control the onset or progression of fibroproliferative conditions in the mouth.

Until this report, the overall sequential chain of events initiated by mechanical strain culminating in the overall cellular responses by gingival fibroblasts has not been reported. In particular, the effect of strain on the overall gene expression profile in gingival fibroblasts has not been disclosed. What we have shown is that although the fundamental mechanism underlying the responses of gingival fibroblasts to strain (involving TGFβ) may have been predicted based on individual pieces of evidence in the literature that have been published using skin and lung fibroblasts, what was not expected was that strain was not able to induce expression of fibrotic mRNAs. Moreover, it was not predicted a priori that gingival fibroblasts were less responsive to TGFβ. The fundamental basis for this reduced response was linked to the lower production of ET-1 by gingival fibroblasts; addition of ET-1 rescued the ability of gingival fibroblasts to respond to TGFβ. These data we feel are important as they suggest that the fundamental basis of scarless tissue repair could be due to the reduced production of ET-1 by gingival fibroblasts; blocking ET-1 signaling might be a viable approach to controlling scarring in response to injury in the skin.
